# Effects of Alcohol on the System

**Published:** 1867-12

**Authors:** 


Effects of Alcohol on the System.—Dr. N. S. Davis (*Chicago Medical Examiner*), in a series of sphygmographic observations in regard to the above, has concluded, in the first place, that the presence of alcohol in the blood diminishes the rapidity of nutrition and disintegration, upon which depend the functions of elimination, calorification, and innervation. In other words, alcohol is a positive organic sedative instead of a diffusive stimulant. In the second place, the alcohol itself acts in the system exclusively as a foreign substance, and as such is ultimately excreted or eliminated without chemical change.

